# Efficient lattice-based revocable attribute-based encryption against decryption key exposure for cloud file sharing

**DOI:** 10.1186/s13677-023-00414-w

**Published:** 2023-03-11

**Authors:** Boxue Huang, Juntao Gao, Xuelian Li

**Affiliations:** 1 School of Telecommunications Engineering, Xidian University, Xi’an, China; 2 School of Mathematics and Statistics, Xidian University, Xi’an, China

**Keywords:** Cloud file sharing, Attribute-based encryption, Dynamic management, Multi-authority, Decryption key exposure

## Abstract

Cloud file sharing (CFS) has become one of the important tools for enterprises to reduce technology operating costs and improve their competitiveness. Due to the untrustworthy cloud service provider, access control and security issues for sensitive data have been key problems to be addressed. Current solutions to these issues are largely related to the traditional public key cryptography, access control encryption or attribute-based encryption based on the bilinear mapping. The rapid technological advances in quantum algorithms and quantum computers make us consider the transition from the tradtional cryptographic primitives to the post-quantum counterparts. In response to these problems, we propose a lattice-based Ciphertext-Policy Attribute-Based Encryption(CP-ABE) scheme, which is designed based on the ring learing with error problem, so it is more efficient than that designed based on the learing with error problem. In our scheme, the indirect revocation and binary tree-based data structure are introduced to achieve efficient user revocation and dynamic management of user groups. At the same time, in order to further improve the efficiency of the scheme and realize file sharing across enterprises, the scheme also allows multiple authorities to jointly set up system parameters and manage distribute keys. Furthermore, by re-randomizing the user’s private key and update key, we achieve decryption key exposure resistance(DKER) in the scheme. We provide a formal security model and a series of security experiments, which show that our scheme is secure under chosen-plaintext attacks. Experimental simulations and evaluation analyses demonstrate the high efficiency and practicality of our scheme.

## Introduction

Cloud file sharing (CFS) has been widely used in current cloud services. According to Gallup’s report, 81% of employees of 60 million full-time employees are choosing to work from home remotely work or mixed work (part-time working from home) by reason of the COVID-19 epidemic. The CFS has the advantages of flexible use and low cost. Employees can access cloud data from any location through any internet device (e.g. mobile phone, tablet, laptop, etc.) to meet flexible remote work needs. At the same time, cloud storage servers can meet the high storage requirements of users and enterprises, and provide low-cost and diversified cloud services. It greatly saves the company’s data storage cost and improves the competitiveness of the enterprise. As a result, CFS services (such as Amazon WorkDocs, iCloud, Dropbox, Google Drive, OneDrive, Mega, etc.) have become the first choice for increasingly competitive individuals and businesses.

As a leading cloud storage platform, MEGA has more than 250 million users, and aims to provide users with end-to-end encryption and information security assurance controlled by users. However, in a recent study [1], Backendal et al. found a significant shortcomings in the platform’s cryptographic architecture. They have carried out five attacks against the RSA encryption algorithm in MEGA, such as RSA key recovery attack, framing attack, integrity attack, etc., to destroy the user data integrity to some extent. Through the RSA key recovery attack, the attacker can recover the RSA private key after 1023 client login attempts, while using quantum cryptanalysis can reduce it to 512 attempts, so that the private key can be recovered more quickly.

Hence, how to protect the user’s data in the cloud from being tampered with, stolen, or illegally accessed by other users has always been a hot research issue. In order to provide fine-grained access control, Attribute-Based Encryption(ABE) has been widely used in various CFS systems [2–6]. In the ciphertext-policy ABE(CP-ABE), the access policy is associated with the ciphertext, and the attribute is associated with the key, which makes CP-ABE more focused on the role-based access control than the key-policy ABE(KP-ABE), Hence CP-ABE is more suitable for CFS systems.

Currently, there are several security and access control issues on the CP-ABE scheme proposed for the CFS system.

$\mathbf {Resistant\ to\ quantum\ attacks:}$ With the further study of quantum computers and quantum attack algorithms, the security of traditional ABE based on bilinear mapping has been seriously challenged. In [7], the author also pointed out that cryptographic algorithms and cryptography-related devices should immediately start to transition to the post-quantum cryptography suite of algorithms. Otherwise, some sensitive documents, such as business secrets, medical records, national security documents and other documents that have a long shelf life can be leaked out since the transition process could take multidecade, by which time the quantum computer may have been mastered by the adversary. Currently, cryptographic algorithms based on the Learning With Error (LWE) problem in lattice are generally considered to be effective against quantum attacks. However, the large parameter size and low efficiency of LWE-based ABE schemes make the cryptographic researcher consider alternative ABE schemes designed based on the Ring Learning With Error (RLWE) problem with smaller parameter size and higer efficiency. Current RLWE-based ABE schemes are deficient in dynamic management of user groups, distributed management and the decryption key exposure resistance. Therefore, the design and security analysis of RLWE-based CP-ABE schemes with multiple properties for CFS system become a challenging issue.

$\mathbf {Dynamic\ management\ of\ user\ groups:}$ In practical applications, the CFS system usually changes users’ data access policy when users’ position or role change, such as revoking the user’s access privileges to specific files and private data. Users’ privileges revocation can be divided into the direct revocation and indirect revocation. In the direct revocation, the data owner can directly revoke the user’s permissions. However, the direct revocation is not applicable in CFS because the data owner must always be online and maintain an up-to-date revocation list at all times. Indirect revocation is suitable for CFS system, in which the attribute authority can periodically broadcasts the key-updating material to users in the CFS system, and users who have not been revoked can update their credentials. However, indirect revocation also faces a problem, that is, the attribute authority needs to generate key update materials for each user in the revocation process. Although Yang et al. [8] and Wang et al. [9] used the binary tree structure to reduce the size of the key update material for each user, this is still a heavy burden for the authorization authority in the CFS system with a large number of users. Therefore, how to implement user revocation securely and efficiently is still an urgent problem.

$\mathbf {Decryption\ key\ exposure\ attacks:}$ In the ABE scheme proposed for CFS system, the user’s decryption key is often used for the decryption of private data. Once the decryption key is exposed, the private data will be opened for the adversary. Hence the key exposure attack is a main secure threat to the CFS system. In the indirect revocation cryptosystems, decryption key exposure attack is a common attack method. The attacker can obtain the user’s long-term private key by calculating the old and leaked decryption key and the update key transmitted from the public channel. This means that the attacker can derive all subsequent decryption keys. In ABE schemes based on the bilinear mapping [10, 11], the author proposed the method of key re-randomization to achieve decryption key exposure resistance. In contrast, the lattice-based CP-ABE scheme has a more complex algebraic structure, and it is more difficult to re-randomize its key. Therefore, how to re-randomize the key of RLWE-based CP-ABE to resist decryption key exposure attack remains challenged.

In addition, the lattice-based multi-authority attribute-based encryption(MA-ABE) scheme could be more suitable for a large-scale CFS system than an ABE scheme with only one authority, because a shared file often has access policies that span multiple trust domains. For example, multiple companies may publish attributes as part of a joint project. If a single authority is employed, one company must be required to cede control to another, which has great limitations in practical applications. Current MA-ABE schemes [12, 13] are designed based on the LWE problems. What we concerns is how to design the RLWE-based MA-ABE schemes with the above desired properties.

### Related work

Some CFS systems with access control encryption or CP-ABE have been proposed. Zhu et al. [14] proposed an efficient temporary access control encryption scheme for cloud services based on a proxy encryption mechanism and cryptographic integer comparison, while extending the power of attribute expression using a dual comparative expression of integer ranges. Zhu et al. [15] proposed an ABE scheme without pairings for CFS systems, and proved the chosen plaintext security in selective ID model. Zhu et al. [16] proposed a new fuzzy authorization scheme based on CP-ABE scheme and OAuth for cloud data access, and realized automatic revocation by updating the TimeSlot attribute when data owner modifies the data. Finally, the security of the scheme is proven under the d-BDHE assumption. Wang et al. [6] proposed an efficient cloud computing encryption scheme based on file-level attributes. Layered files are encrypted with an integrated access structure. However, the above ABE schemes are all based on bilinear pairing and do not have the ability to resist quantum attacks.

Considering the hardness of lattice problems in post-quantum cryptography, researchers begin to construct lattice-based ABE schemes. Zhang et al. [17] first constructs a CP-ABE scheme based on the LWE assumption. However, due to the introduction of default properties, the parameter size of the overall scheme increases dramatically and the efficiency is low. In order to solve this problem, chen et al. [2] uses a small policy matrix to reduce the cumulative error and improve the efficiency of the scheme. At the same time, they also proposed a new resource sharing framework combined with the ABE scheme. Chen et al. [18] improved on the basis of [17], and implemented a large universe CP-ABE scheme using the full-rank differences function, which can obtain greater efficiency. To further improve efficiency, Gür et al. [19] constructs and implements the CP-ABE scheme from lattices under the RLWE assumption. They employ Gaussian sampling for bigger bases of the gadget matrix, which reduces execution times and storage requirements.

In order to solve the user dynamic management problem in the CFS system, Ibraimi et al. [20] proposed a revocable CP-ABE scheme, but this scheme requires a trusted cloud service provider and requires it to be online for a long time. This does not meet actual needs. Sahai et al. [21] proposed a CP-ABE scheme with revocable storage, which allows a cloud Server to update the ciphertext, but this scheme adds many additional time attributes, resulting in bigger public key and ciphertext. Li et al. [3] proposed an efficient CP-ABE scheme with policy update and file update functions based the CFS system, which effectively reduces the communication and storage cost of the client.

In order to prevent decryption key exposure attacks in CFS systems, Xu et al. [10] proposes a CP-ABE scheme with decryption key exposure resistance. Xu et al. [22] proposed a CP-ABE scheme with decryption key exposure resistance based on cloud storage system, which can effectively deal with both secret key revocation for corrupted users and accidental decryption key exposure for honest users. However, the above revocable CP-ABEs are all designed based on bilinear pairing, and they cannot effectively resist quantum attacks. Wang et al. [9] and yang et al. [23] proposed two LWE-based revocable CP-ABE schemes. However, the public key size of scheme [9] is large, and the revocation workload of scheme [23] increases linearly with the increase of the number of system users, which makes the efficiency and scalability of the two schemes low. Takayasu et al. [24] and Dong et al. [25] proposed lattice-based IBE scheme and KP-ABE scheme for decryption key exposure resistance, respectively. But in the lattice-based CP-ABE scheme, which is more suitable for CFS system, there is no formal solution.

In order to further improve the efficiency of the CFS system, reduce the workload of a single AA on the enterprise side, and realize access policies across multiple trust domains at the same time, Chase et al. [26] proposed the multi-attribute authority ABE scheme for the first time. The scheme includes a central authority and multiple attribute authorities, and the attributes in the system are jointly managed by multiple attribute authorities. Subsequently, Rouselakis et al. [27] proposed an MA-ABE scheme based on prime order bilinear groups to further improve the efficiency. To deal with quantum attacks, Zhang et al. [13] proposed lattice-based MA-ABE for the first time. However, since this scheme is based on the assumption of learning with errors, the efficiency of this scheme is low.

### Technical challenge

Current lattice-based CP-ABE schemes for CFS system, especialy for LWE-based CP-ABE scheme, suffer from large parameter size, low efficiency or various cryptographic attacks, such as decryption key exposure attack, collusion attack etc.. When we design an RLWE-based CP-ABE with multiple properties, such as, multiple authorities, decryption key exposure resistance, collusion resistance, user group’s dynamic management, attribute revocation etc., several technical chanllenges loom over us. Specifically, when designing an RLWE-based CP-ABE with multiple authorities and decryption key exposure resistance, we have to consider a new type of adversary who masters more information on the private keys of some attributes. The adversary could corrupt some AAs to get some information on the private keys of some attributes. Simultaneously, the adversary can get some addtional infomation leaked out from the uncorrupted AAs due to the decryption key exposure. As the adversary gains more information, the security model becomes more complex. Hence the security proof in our scheme is more difficult than that in the scheme with one single authority, or the scheme with only decryption key exposure resistance. Furthermore, in the traditional ABE schemes based on bilinear pairing, the resistance to decryption key exposure attack is simply implemented by introducing a random number in the decryption key update. However, the lattice-based CP-ABE scheme is usually designed based on a more complex algebraic structure, which makes the original method(by only introducing a random number in the re-randomization process) for decryption key update invalid. Therefore, in order to get the new RLWE-based MA-ABE suitable for the secure CFS system, we need to define new security model, specify the limits of the new adversary and develop new technique to resist the decryption key exposure attack.

### Our contribution

In this paper, we designed an RLWE-based Revocable and Multi-authority CP-ABE scheme (RM-CP-ABE) with decryption key exposure resistance for quantum secure CFS system, whose features includes protection of sensitive data from privacy leakage and quantum attacks, dynamic management of user groups, distributed architecture, decryption key exposure resistance, etc. Our work is summarized as follows:

- Many existing CFS systems only provide role-based access control and are not resistant to quantum attacks. We design a CP-ABE scheme based on RLWE to achieve more fine-grained access control and CFS privacy data security under quantum attacks. In order to resist the decryption key attack, we propose a new method applicable to lattice algebraic structure for re-randomizing the key to ensure that the information of the user’s private key will not be leaked after the decryption key is exposed. To implement user revocation, we apply a binary tree-based data structure to reduce the cost of key update from linear to logarithmic.
- In order to further improve the system efficiency and realize cross-enterprise file sharing, we implement a multi-authority CP-ABE scheme with a distributed architecture by applying the shamir threshold secret sharing technique. At the same time, in order to ensure the security of private data under collusion attacks by malicious users and corrupt authorities, we provide a specific security model and prove that our scheme is selectively secure under the assumption of error learning.
- Finally, we implement our scheme and evaluate its key generation, user revocation, encryption, and decryption algorithm time overhead and storage overhead in comparison with related schemes. The simulation show that our scheme has high efficiency.

## Preliminaries

### Notations

In this paper, we denote a cyclotomic ring $\mathcal {R}=\mathbb {Z}\left[ x \right] /\left\langle x^{n}+1 \right\rangle$, where each element is a polynomial with integer coefficients and degree at most $n-1$. For an integer $q\ge 2$, we let $\mathcal {R}_{q}=\mathcal {R}/q\mathcal {R}$ be a ring in which arithmetic operations on polynomial coefficients are performed modulo *q*, and the coefficients are integers in the interval $\left( \left\lfloor -q/2 \right\rfloor ,\left\lfloor q/2 \right\rfloor \right)$. $\mathcal {R}_{q}^{1\times m}$, $\mathcal {R}_{q}^{m}$, and $\mathcal {R}_{q}^{m\times m}$ represent row vectors, column vectors, and matrices consisting of elements in $\mathcal {R}_{q}$. We define $Tran_{V\rightarrow M}$ as a function that maps from vector $\textbf{a}\in \mathcal {R}_{q}^{m}$ to matrix $\textbf{A}\in \mathbb {Z}_{q}^{m\times n}$. In detail, we expand the coefficients of each polynomial element in vector $\textbf{a}$ to an *n*-dimensional row vector in $\mathbb {Z}_{q}$. Similarly, we define $Tran_{M\rightarrow V}$ as a function that maps from matrix $\textbf{A}\in \mathbb {Z}_{q}^{m\times n}$ to vector $\textbf{a}\in \mathcal {R}_{q}^{m}$, which can be regarded as the inverse process of $Tran_{V\rightarrow M}$. In this paper, we utilize a full-rank difference (FRD) function $\textrm{H}$ that maps a random element in $\mathcal {R}_{q}$ associated with an attribute or time to a matrix in $\textbf{Z}_{q}^{n \times n}$.

#### Lemma 1

[28] For positive integers *q*, *n* with $q>2$, the FRD function has the following properties:

- For any $a,b \in \mathcal {R}_{q}$ and $a\ne b$, the matrix $\left( \textrm{H}\left( \textrm{a} \right) -\textrm{H}\left( \textrm{b} \right) \right)$ is full rank.
- H is computable in polynomial time $n\,\log \,q$.

#### Lemma 2

$(Leftover\ Hash\ Lemma)$ Let *q* be a positive integer, $m=O(\log _{2}q)$ and let $\textbf{S}\leftarrow \left\{ -1,1 \right\} ^{m\times m}$, $\left\{ \textbf{A,B} \right\} \leftarrow \mathcal {R}_{q}^{1\times m}$. Then for all $e \in \mathcal {R}_{q}^{m}$, we have $\left( \textbf{A,AS,e}^{\varvec{\top }}\textbf{S} \right)$ is statistically close to $\left( \textbf{A,B,e}^{\varvec{\top }}\textbf{S} \right)$.

### Lattice

A full rank lattice $\Lambda$ is a discrete additive subgroup of $\mathbb {R}^{n}$. Given a positive intger *n* and a basis $\textbf{B}= \left\{ b_{1},...,b_{n} \right\} \subseteq \mathbb {R}^{n}$, the lattice $\Lambda$ can be represented as

$$\begin{aligned} \Lambda = \mathcal {L}\left( \textbf{B} \right) =\left\{ \textbf{Bx}=\sum _{i=1}^{n}x_{i}\textbf{b}_{i}\mid \textbf{x}\in \mathbb {Z}^{n} \right\} . \end{aligned}$$

For an uniformly randomly matrix $\textbf{A}\in \mathbb {Z}_{q}^{n\times m}$ and a vector $\textbf{u}\in \mathbb {Z}_{q}^{n}$, the *q*-ary lattice $\Lambda _{q}^{\perp }(\textbf{A})$ and its coset $\Lambda _{q}^{\textbf{u}}(\textbf{A})$ is denoted by

$$\begin{aligned} \Lambda _{q}^{\perp }(\textbf{A})=\left\{ \textbf{x}\in \mathbb {Z}^{\textbf{m}}\mid \textbf{Ax}=0 \mod \ q \right\} , \end{aligned}$$

$$\begin{aligned} \Lambda _{q}^{\textbf{u}}(\textbf{A})=\left\{ \textbf{x}\in \mathbb {Z}^{\textbf{m}}\mid \textbf{Ax}=\textbf{u} \mod \ q \right\} . \end{aligned}$$

$\mathbf {Discrete\ Gaussian\ Distribution}$: Given a lattice $\Lambda$ with a parameter $\sigma \in \mathbb {R}$ and a center vector $\varvec{c}\in \mathbb {R}^{n}$, we denote the *n*-th dimensional discrete Gaussian distribution by

$$\begin{aligned} D_{\Lambda ,\varvec{c},\sigma }=\frac{\rho _{\varvec{c}, \sigma } \left( \varvec{x} \right) }{\sum _{\varvec{z}\in \Lambda } \rho _{\varvec{c}, \sigma } \left( \varvec{z} \right) } \end{aligned}$$

where $$\varvec{x}\in \Lambda$$ and $$\rho _{\varvec{c}, \sigma } = \text {exp}\left( -\pi \left\| \varvec{x-c} \right\| ^2/2\sigma ^{2} \right)$$.

#### Definition 1

$(\mathbf {Ring\ Learning\ With\ Errors})$ The Ring Learning With Errors (RLWE) hardness assumption holds if for any PPT adversary $\mathcal {A}$ we have

$$\begin{aligned} \left| \text {Pr}\left[ \mathcal {A}\left( a_{i},a_{i}s+e_{i} \right) -\mathcal {A}\left( a_{i},u_{i} \right) \right] \right| < negl\left( \lambda \right) \end{aligned}$$

where $\left\{ s,a_{i} \right\} \leftarrow \mathcal {R}_{q}$, $e_{i}\leftarrow D_{\mathcal {R},\sigma }$, $i=0,...,n$ and the probability is taken over the choice of the random coins by the PPT adversary $\mathcal {A}$.

### Lattice trapdoors

Lattices with trapdoors are described in [8, 19, 29], and they are statistically indistinguishable from randomly chosen lattices. But trapdoors have some extra information that can be used for efficient solution to the hard problem on lattices. In this paper, we utilize the trapdoor sampler from [19, 29]. The trapdoor sampler contains $\text {TrapGen}$ algorithm, $\text {SamplePre}$ algorithm and two expansion algorithms: $\text {SampleLeft}$ algorithm and $\text {SampleRight}$ algorithm, which are given as follows.

- $\textbf{TrapGen}\left( \lambda \right) \rightarrow \left( \textbf{A},\textbf{T}_{\textbf{A}} \right)$: The lattice generation algorithm takes the security parameters $\lambda$ and $\sigma$, modulus *q*, and a polynomial degree *n* as input. Then it outputs a vector $\textbf{A} \in \mathcal {R}^{1 \times m}_q$ together with a trapdoor $\textbf{T}_{\textbf{A}}$.
- $\textbf{SamplePre}\left( \textbf{A},\textbf{T}_{\textbf{A}},u,\sigma ,\sigma _{s} \right) \rightarrow \textbf{s}$: The presampling algorithm takes a vector $\textbf{A}\in \mathcal {R}_{q}^{1 \times m}$, a trapdoor $\textbf{T}_{\textbf{A}}$, a target $u \in \mathcal {R}$, and a pair of parameters $\left( \sigma , \sigma _{s} \right)$ as input. It outputs a vector $\textbf{s} \leftarrow D_{\mathcal {R}_{q}^{m}}$ such that $\textbf{A}\cdot \textbf{s}=u$.
- $\textbf{SampleRight}\left( \textbf{A},\textbf{B},\textbf{T}_{\textbf{A}},u \right) \rightarrow \textbf{s}$: The algorithm takes as input a pair of vectors $\left( \textbf{A},\textbf{B} \right) \in \mathcal {R}_{q}^{1 \times m}\times \mathcal {R}_{q}^{1 \times m}$, a trapdoor $\textbf{T}_{\textbf{A}}$ and target $u \in \mathcal {R}$, and outputs a vector $\textbf{s} \leftarrow D_{\mathcal {R}_{q}^{2m}}$ such that $\left( \textbf{A}\mid \textbf{B} \right) \cdot \textbf{s}=u$.
- $\textbf{SampleLeft}\left( \textbf{A},\textbf{B},\textbf{T}_{\textbf{B}},\textbf{S},u \right) \rightarrow \textbf{s}$: The algorithm takes as input a pair of vectors $\left( \textbf{A},\textbf{B} \right) \in \mathcal {R}_{q}^{1 \times m}\times \mathcal {R}_{q}^{1 \times m}$, a trapdoor $\textbf{T}_{\textbf{B}}$, a matrix $\textbf{S}\leftarrow \left( \pm 1 \right) ^{m\times m}$ and target $u \in \mathcal {R}$, and outputs a vector $\textbf{s} \leftarrow D_{\mathcal {R}_{q}^{2m}}$ such that $\left( \textbf{A}\mid \textbf{A}\textbf{S}+\textbf{B} \right) \cdot \textbf{s}=u$.

#### Definition 2

$(\mathbf {Well-Sampledness\ of\ Vector})$[12] The lattice generation algorithm is said to satisfy the well-sampledness of vector property if for any $\lambda \in \mathbb {N}$, there exists a negligible function $negl\left( \lambda \right)$ such that the vector $\textbf{A}$ is $negl\left( \lambda \right)$-close to uniform, where $\textbf{A}$ is generated by $\textbf{TrapGen}\left( \lambda \right)$.

#### Definition 3

$(\mathbf {Well-Sampledness\ of\ Preimage})$ [12] The presampling algorithms and two expansion algorithms are said to satisfy the well-sampledness of preimage property if for any $\lambda \in \mathbb {N}$, there exists a negligible function $negl\left( \lambda \right)$ such that the distribution of the vector $\textbf{s}_{\varvec{1}}$ and $\textbf{s}_{\varvec{2}}$ are $negl\left( \lambda \right)$-close to $D_{\mathcal {R}_{q}^{m}}$ and $D_{\mathcal {R}_{q}^{2m}}$ respectively, where $\textbf{s}_{\varvec{1}}$ is sampled by $\textbf{SamplePre}\left( \textbf{A},\textbf{T}_{\textbf{A}},u,\sigma ,\sigma _{s} \right)$ algorithm and $\textbf{s}_{\varvec{2}}$ is sampled by one of the two expansion algorithms with the same parameters.

### The binary-tree data structure

The binary tree-based data structure is mainly used to reduce the calculation cost of generating key-updating material from linear to logarithmic. In the binary tree structure, each user is associated with the leaf node. Except for leaf nodes, each node has two children nodes. Let *Path*(*v*) be the node set on the path from the root node of the tree to node *v*, and $(v_{l},v_{r})$ be the left child node and the right child node of node *v* respectively if *v* is a non-leaf node. When a user $v_{i}$ is revoked at time $t_{i}$, add the user to the revocation list *rl*, and then the subset covering algorithm $\text {KUNodes}(st,rl,t)$ [30] is run to fatch the minimum set of key-updating materials related to non-revoked users. The $\text {KUNodes}(st,rl,t)$ algorithm is shown as Algorithm 1.

**Figure:**
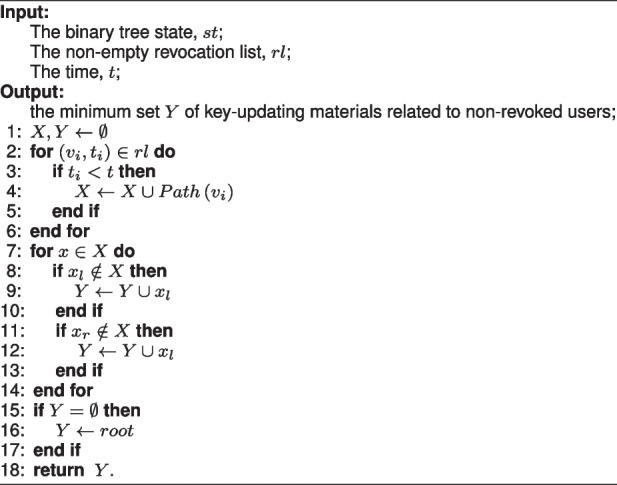
**Algorithm 1** $$\text {KUNodes}(st,rl,t)$$

## System framework

In this section, we present the system model and threat model of CFS with our new RM-CP-ABE scheme. We then provide a formal security model to simulate the adversary’s attacks in the model.

### RM-CP-ABE scheme

#### Definition 4

There are eight algorithms in our RM-CP-ABE scheme: GlobalSetup, AuthSetup, KeyGen, KeyUpdate, DKGen, Enc, Dec, and Rev, with associated attribute space $\mathcal {U}$, user space $\mathcal {I}$, time space $\mathcal {T}$, and message space $\mathcal {M}$. The algorithms are defined as follows:

$\text {GlobalSetup}(\lambda , N)\rightarrow pp$: The global setup algorithm takes as input the security parameter $\lambda$ and the number of authorities *N*, and outputs the public parameters *pp*.

$\text {AuthSetup}(pp,\theta )\rightarrow (pk_{\theta }, msk_{\theta })$: The authority setup algorithm takes as input the public parameters *pp*, and a number $\theta$ to represent the $\theta$th authority $AA_{\theta }$, and outputs a pair of $\left( pk_{\theta }, msk_{\theta } \right)$ as the public key and master secret key.

$\text {KeyGen}(pk_{\theta }, msk_{\theta },st,S)\rightarrow sk_{\theta }$: The key generation algorithm takes as input the public key $pk_{\theta }$, master secret key $msk_{\theta }$, a state *st* and a set of attributes *S*, and outputs the secret key $sk_{\theta }$ associated with $AA_{\theta }$.

$\text {KeyUpdate}(pk_{\theta }, msk_{\theta },rl,st,t)\rightarrow ku_{t}$: The key update algorithm inputs the public key $pk_{\theta }$, the master secret key $msk_{\theta }$, a revocation list *rl*, and a state *st*, and a time *t* and outputs an update key $ku_{t}$.

$\text {DKGen} \left( \{sk_{\theta }, ku_{\theta }\}_{\theta \in N} \right) \rightarrow dk$: The decryption key generation algorithm takes the secret key $\{sk_{\theta }\}_{\theta \in N}$ and the key update $\{ku_{\theta }\}_{\theta \in N}$ as input. If the user is not revoked, the algorithm outputs the decryption key. Otherwise, it outputs $\perp$.

$\text {Enc} \left( pp, \{pk_{\theta }\}_{\theta \in N}, \mu ,\mathbb {W} \right) \rightarrow CT$: The encryption algorithm takes as input the public parameters *pp*, the public key $\{pk_{\theta }\}_{\theta \in N}$, a message $\mu$, and an access policy $\mathbb {W}$ and outputs a ciphertext *CT*.

$\text {Dec} \left( CT, dk \right) \rightarrow \mu$: The decryption algorithm takes as input the ciphertext *CT* and the decryption key *dk*. If the attribute set *S* corresponding to the decryption key *dk* satisfies the access policy $\mathbb {W}$, the algorithm outputs a message $\mu$. Otherwise it outputs $\perp$.

$\text {Rev} \left( rl, id, t \right) \rightarrow rl$: The revocation algorithm takes as input the revocation list *rl*, an user’s global identity *id* to be revoked and a time *t*, and outputs the updated revocation list *rl*.

#### Definition 5

$(\textbf{Correctness})$ The correctness of RM-CP-ABE requires that for every security parameter $\lambda \in \mathbb {N}$, every message $\mu$, every access policy $\mathbb {W}$, and every set of attribute *S* which satisfy the access policy $\mathbb {W}$ it holds that

$$\begin{aligned} \textrm{Pr}\left[ \mu ' = \mu \mid \begin{array}{c} pp \leftarrow \text {GlobalSetup}(\lambda , N) \\ (pk_{\theta }, msk_{\theta })\leftarrow \text {AuthSetup}(pp,\theta )\\ sk_{\theta } \leftarrow \text {KeyGen}(pk_{\theta }, msk_{\theta },st,S) \\ ku_{t} \leftarrow \text {KeyUpdate}(pk_{\theta }, msk_{\theta },rl,st,t) \\ dk \leftarrow \text {DKGen} \left( \{sk_{\theta }, ku_{\theta }\}_{\theta \in N} \right) \\ CT \leftarrow \text {Enc} \left( pp, \{pk_{\theta }\}_{\theta \in N}, \mu ,\mathbb {W} \right) \\ \mu ' \leftarrow \text {Dec}\left( CT, dk \right) \end{array}\right] =1 \end{aligned}$$

### System model

We propose a secure CFS system framework based on the RM-CP-ABE scheme, which mainly includes four typical parties described as follows:

$\mathbf {Attribute\ Authorities(AAs)}$: AAs are authorization management entities set up by the enterprise side independently of the cloud server, responsible for initializing the system and broadcasting system parameters to other entities, and maintaining the access credentials of users in the system. In addition, every update cycle, the enterprise will revoke users who have lost access by publicly broadcasting key-updating materials. AAs consists of a central authority(CA) and several attribute authorities. This distributed architecture not only reduces the workload of a single attribute authority, but also enhances the scalability of the system and realizes CFS across enterprises.

$\mathbf {Data\ Owners(DOs)}$: DOs are the initiators and owners of shared files. It encrypts files through the client, sets corresponding access policies, and finally uploads the encrypted files to the cloud.

$\mathbf {Data\ Users(DU)}$: DUs are users of shared files. Users with access rights can decrypt the file through the client to read the file content.

$\mathbf {Cloud\ Service\ Provider(CSP)}$: CSP provides cloud-based file storage and file sharing services for CFS systems.

**Fig. 1:**
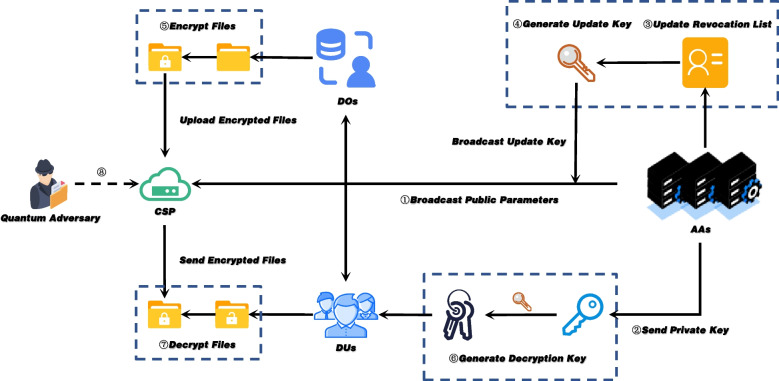
System Model

**Fig. 2:**
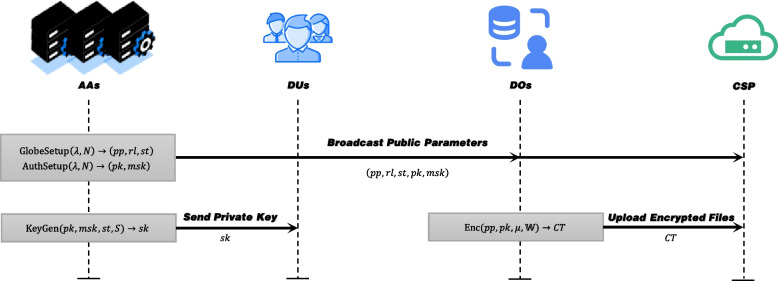
System Initialization and Files sharing

As shown in Fig. 1, the workflow of the CFS system based on the RM-CP-ABE scheme can be divided into the following four steps:

System initialization: Fig. 2 shows the CFS system initialization phase. CA runs $\text {GlobalSetup}(\lambda , N)\rightarrow pp$ and each AA runs $\text {AuthSetup}(pp,\theta )\rightarrow (pk_{\theta }, msk_{\theta })$ to generate system parameters, then broadcast the $(pp,pk_{\theta })$ to DOs and CSP(see Fig. 1 ①). AAs runs $\text {KeyGen}(pk_{\theta }, msk_{\theta },st,S)\rightarrow sk_{\theta }$ to generate and send private key to the corresponding user (see Fig. 1 ②).

File sharing: As shown in Fig. 2, DOs runs $\text {Enc} \left( pp, \{pk_{\theta }\}_{\theta \in N}, \mu ,\mathbb {W} \right) \rightarrow CT$ to encrypt the files and upload them to CSP in the file sharing phase(see Fig. 1 ⑤).

**Fig. 3:**
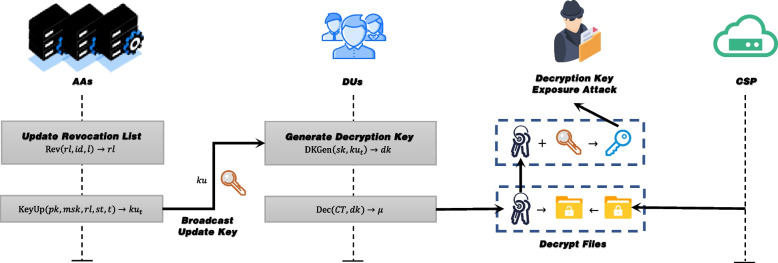
User Management and Files Decryption

User management: As shown in Fig. 3, in the user management phase, the AAs runs $\text {Rev} \left( rl, id, t \right) \rightarrow rl$ to add users who have lost access rights to the revoked list (see Fig. 1 ③), and runs algorithm $\text {KeyUpdate}(pk_{\theta }, msk_{\theta },rl,st,t)\rightarrow ku_{t}$ to generate and broadcast the updated key during the key update period (see Fig. 1 ④).

Files Decrytion: As shown in Fig. 3, in the files decrytion phase, the DUs with access rights first receives the update key of the current period and runs $\text {DKGen} \left( \{sk_{\theta }, ku_{\theta }\}_{\theta \in N} \right) \rightarrow dk$ to generate a temporary decryption key(see Fig. 1 ⑥). Then DUs runs $\text {Dec} \left( CT, dk \right) \rightarrow \mu$ to decrypt and read the files contents(see Fig. 1 ⑦).

### Threat model

In our system, the CSP is honest and curious, that is, it follows the protocol, but tries to get as much sensitive information in the files as possible through observations. In addition, data stored in the cloud is more vulnerable to some adversary, such as hackers. In order to prevent the semi-trusted CSP and various adversaries (including quantum adversaries, see Fig. 1⑧) from getting the sensitive information, we introduce RM-CP-ABE based on RLWE to ensure the security of CFS systems in the post-quantum era. Furthermore, in the threat model, we allow collusion attacks between revoked users and unauthorized users to attempt to obtain useful information in encrypted files. At the same time, the attacker can also launch some attacks by means of the corrupted AAs. Hence, collusion attacks between the corrupted AA and the revoked users pose a serious threat to the system’s security. This forces our RM-CP-ABE scheme to have the ability to resist collusion attacks. Furthermore, as shown in Fig. 3, for the practical decryption key exposure attack in indirectly revocable cryptosystems, our scheme is also required to have the ability to resist decryption key exposure. For the various types of attacks that may be encountered in the real world, we present the following security model.

### Security model

#### Definition 6

We define the selective IND-CPA security with Decryption Key Exposure Resistance(DKER) for RM-CP-ABE scheme by the following game between an adversary $\mathcal {A}$ and a challenger $\mathcal {C}$.

$\mathbf {Init.}$ The adversary $\mathcal {A}$ chooses a challenge access structure $\mathbb {W}^{*}$ and a time $t^{*}$, and give them to $\mathcal {C}$. The challenge access policy contains two subsets, the positive attribute set $\mathbb {W}^{+}$ and the negative attribute set $\mathbb {W}^{-}$, where $\mathbb {W}^{+}\cap \mathbb {W}^{-}=\emptyset$. Then, $\mathcal {A}$ chooses a set of corrupted AAs $\textbf{Cor}$ and publishes the set.

$\mathbf {Setup.}$ $\mathcal {C}$ calls $\text {GlobalSetup}$ algorithm to acquire and send *pp* to $\mathcal {A}$. Then, $\mathcal {C}$ performs the $\text {AuthSetup}$ algorithm to obtain and send their corresponding public and private key pairs $\left\{ pk_{\theta }, msk_{\theta } \right\} _{\theta \in N}$. For the set of uncorrupted authorities, $\mathcal {C}$ sends the corresponding public key $\left\{ pk_{\theta } \right\} _{\theta \notin \textbf{Cor}}$. Otherwise, $\mathcal {C}$ sends the corresponding public-private key pair $\left\{ pk_{\theta }, msk_{\theta } \right\} _{\theta \in \textbf{Cor}}$.

$\mathbf {Key\ Query.}$ $\mathcal {A}$ is allowed to adaptively make the following query to $\mathcal {C}$ on the attribute set $\textbf{S}$:

- $$sk_{\theta } \leftarrow \mathcal {A}^{\mathcal {O}^{KeyGen\left( \cdot \right) }}\left( \textbf{S} \right)$$ for all $$\theta \notin \textbf{Cor}$$.
- $$ku_{\theta ,t} \leftarrow \mathcal {A}^{\mathcal {O}^{KeyUpdate\left( \cdot \right) }}\left( t,rl,st \right)$$ for all $$\theta \notin \textbf{Cor}$$.
- $$dk\leftarrow \mathcal {A}^{\mathcal {O}^{DKGen\left( \cdot \right) }}\left( sk_{\theta },ku_{\theta ,t } \right)$$.
- $$\left( rl,st \right) \leftarrow \mathcal {A}^{\mathcal {O}^{Rev\left( \cdot \right) }}\left( rl,id,t \right)$$.

When the adversary has access to the oracle, there are some restrictions as follows, and these restrictions are divided into two cases:

$\mathbf {Case\ I.}$ $\mathcal {O}^{KeyGen\left( \cdot \right) }$ can not be queried with the attribute set $\textbf{S}$ satisfying $\mathbb {W}^{*}$.

$\mathbf {Case\ II.}$ If $\mathcal {O}^{KeyGen\left( \cdot \right) }$ was queried with the attribute set $\textbf{S}$ satisfying $\mathbb {W}^{*}$, then $\mathcal {O}^{Rev\left( \cdot \right) }$ must be queried on this user *id* before time $t^{*}$.

In addition, both cases are held to the following restrictions:

- $\mathcal {O}^{KeyUpdate\left( \cdot \right) }$ and $\mathcal {O}^{Rev\left( \cdot \right) }$ are only allowed to query in non-decreasing time sequence.
- If $\mathcal {O}^{KeyUpdate\left( \cdot \right) }$ was queried at the time *t*, $\mathcal {O}^{Rev\left( \cdot \right) }$ can not be queried at the time *t*.
- $\mathcal {O}^{DKGen\left( \cdot \right) }$ cannot be queried for the decryption key *dk* corresponding to the user whose attribute set $\textbf{S}$ satisfies $\mathbb {W}^{*}$ or the user that has been revoked.

$\mathbf {Challenge.}$ $\mathcal {A}$ chooses two equal-length message $\mu _{1},\mu _{2} \in \mathcal {M}$ and sends them to $\mathcal {C}$. Then $\mathcal {C}$ randomly flips a coin $b\leftarrow \left\{ 0,1 \right\}$ and replies $CT \leftarrow \text {Enc} \left( pp, \{pk_{\theta }\}_{\theta \in N}, \mu _{b} ,\mathbb {W} \right)$ to $\mathcal {A}$.

$\mathbf {Guess.}$ The adversary $\mathcal {A}$ outputs the guessed $b'$ for *b*.

Adversary $\mathcal {A}$’s advantage in this game is defined as follows:

$$\begin{aligned} Adv_{\mathcal {A}}^{IND-CPA}\left( \lambda \right) =\left| \textrm{Pr}\left[ b=b' \right] -1/2 \right| . \end{aligned}$$

#### Definition 7

$(\mathbf {sIND-CPA\ in\ RM-CP-ABE})$ An RM-CP-ABE scheme is the selective IND-CPA security with DKER, if for any PPT adversary $\mathcal {A}$, there exists a negligible function $\text {negl}(\cdot )$ such that the advantage of adversary $Adv_{\mathcal {A}}^{IND-CPA}\left( \lambda \right) \le \text {negl}(\cdot )$.

## Construction

In this section, we provide the detailed construction of our RM-CP-ABE scheme for access structure represented by an AND gate over positive and negative attributes. The positive attribute in the access policy requires that the user must have this attribute to decrypt the corresponding ciphertext. On the other hand, the negative attribute in the access policy requires that the attribute set of the decrypting user cannot contain this attribute. The scheme supports *N* attribute authorities in the system, and efficient user revocation. The details are given below.

$\text {GlobalSetup}(\lambda , N)\rightarrow (pp,st,rl)$: The algorithm inputs a security parameter $\lambda$ and the number of attribute authorities $N\in \mathbb {N}$. It first chooses a set of positive integers *q*, *n*, *m*. Next, it samples a vector $\textbf{y}\in \mathcal {R}_{q}^{1\times m}$ and an element $\beta \in \mathcal {R}_{q}$. Furthermore, it specifies an FRD function H. Finally, it outputs the public parameters

$$\begin{aligned} pp=\left( q, n, m, \sigma , \sigma _{s}, \textbf{y}, \beta , \textrm{H}\right) . \end{aligned}$$

$\text {AuthSetup}(pp,\theta )\rightarrow (pk_{\theta }, msk_{\theta })$: The algorithm takes the public parameters and the number of the attribute authority as input. Each attribute authority $AA_{\theta }$ runs the algorithm and generates a vector-trapdoor pair $\left( \textbf{A}_{\theta }, \textbf{T}_{\textbf{A}_{\varvec{\theta }}}\right) \leftarrow \text {TrapGen}(\lambda )$, and a pair of vectors $\left( \textbf{B}_{\theta , i}^{+}, \textbf{B}_{\theta , i}^{-}\right) \leftarrow \mathcal {R}_{q}^{1 \times m}$. Next, it samples vectors $\left\{ \textbf{D}_{\theta },\textbf{E}_{\theta } \right\} \leftarrow \mathcal {R}_{q}^{1 \times m}$ and outputs the public key and master secret key for the authority $\theta$

$$\begin{aligned} pk_{\theta }=\left( \textbf{A}_{\theta },\left\{ \textbf{B}_{\theta , i}^{+} ; \textbf{B}_{\theta , i}^{-}\right\} _{i \in \left[ l_{\theta }\right] }, \textbf{D}_{\theta },\textbf{E}_{\theta } \right) , \,\,\, msk_{\theta }=\textbf{T}_{\textbf{A}_{\varvec{\theta }}}. \end{aligned}$$

$\text {KeyGen}(pk_{\theta }, msk_{\theta },st,\textbf{S})\rightarrow sk$: The algorithm takes the public key $pk_{\theta }$, the master secret key $msk_{\theta }$ of $AA_{\theta }$, the state *st* and a user’s attribute set $\textbf{S}\subseteq l_{\theta }$ as input, where $l_{\theta }$ is the set of attributes managed by the $AA_{\theta }$. It then proceeds as follows.

1. CA takes a polynomial $P(x)=\beta +\sum _{i=1}^{N-1} a_{i} x^{i}$ of degree $N-1$ for $a_{i}\leftarrow \mathcal {R}_{q}$, computes $\beta _{\theta }=P\left( \theta \right)$ and assigns $\beta _{\theta }$ to $AA_{\theta }$.
2. For each $i\in l_{\theta }$, $AA_{\theta }$ chooses a vector $\textbf{k}_{\theta , i} \leftarrow D_{\mathcal {R}_{q}^{m}, \sigma _{s}}$. If $i\in \textbf{S}$, it computes $u_{\theta , i} \leftarrow \textbf{B}_{\theta ,i}^{+} \textbf{k}_{\theta , i}$, else, it computes $u_{\theta , i} \leftarrow \textbf{B}_{\theta ,i}^{-} \textbf{k}_{\theta , i}$.
3. For each node $\alpha \in Path(id)\setminus Path(id')$, if the node is empty, $AA_{\theta }$ randomly chooses $u_{A_{\theta }, \alpha , 2} \leftarrow R_{q}$ and store it in the node, where *id* denotes the user’s identity who generated the secret key and $id'$ denotes the user’s identity who has been revoked. It implies that the nodes $\alpha \in Path(id')$ corresponding to the revoked user identity $id'$ have been invalidated, and the $AA_{\theta }$ does not need to generate secret keys corresponding to these potentially shared nodes for subsequent users.
4. $AA_{\theta }$ calculates $u_{A_{\theta }, \alpha , 1}=\beta _{\theta }-u_{A_{\theta , \alpha , 2}}-\sum _{i \in l_{\theta }} u_{\theta , i}$ and samples
  $$\begin{aligned} \textbf{k}_{\textbf{A}_{\theta }, \alpha , 1} \leftarrow \text{ SamplePre }\left( \textbf{A}_{\theta }, \textbf{T}_{\textbf{A}_{\varvec{\theta }}}, u_{A_{\theta }, \alpha , 1}, \sigma , \sigma _{s}\right) .\end{aligned}$$
5. $AA_{\theta }$ samples $\textbf{k}_{\textbf{A}_{\theta },3}\leftarrow D_{\mathcal {R}_{q}^{2m},\sigma _{s}}$ and calculates $u_{A_{\theta }, 3}\leftarrow \left( \textbf{A}_{\theta }\mid \textbf{E}_{\theta } \right) \textbf{k}_{\textbf{A}_{\theta },3}$.
6. $AA_{\theta }$ chooses $u_{A_{\theta }, 4}\leftarrow \mathcal {R}_{q}$ and stores it.
7. Then $$AA_{\theta }$$ samples
  $$\begin{aligned} \textbf{k}_{\textbf{A}_{\theta },4} \leftarrow \text{ SamplePre }\left( \textbf{A}_{\theta }, \textbf{T}_{\textbf{A}_{\varvec{\theta }}}, u_{A_{\theta },4}-u_{A_{\theta },3}, \sigma , \sigma _{s}\right) \end{aligned}$$.
8. Finally, it outputs the secret key
  $$\begin{aligned} sk_{\theta }=\left( \left\{ \textbf{k}_{\textbf{A}_{\theta }, \alpha , 1} \right\} _{\alpha \in Path(id)\setminus Path(id')}, \left\{ \textbf{k}_{\theta , i} \right\} _{i\in S},\textbf{k}_{\textbf{A}_{\theta },3},\textbf{k}_{\textbf{A}_{\theta },4} \right) \end{aligned}$$

$\text {KeyUpdate}(pk_{\theta }, msk_{\theta },rl,st,t)\rightarrow ku$: Given the public key $pk_{\theta }$, the master secret key $msk_{\theta }$ of $AA_{\theta }$, the revocation list *rl*, the state *st* and a revocation time *t*, the algorithm proceeds as follows.

1. It samples $r_{t}\leftarrow D_{\mathcal {R}_{q},\sigma _{s}}$ and samples $\textbf{k}_{\textbf{A}_{\theta },5}\leftarrow D_{\mathcal {R}_{q}^{2m},\sigma _{s}}$.
2. It calculates $$u_{A_{\theta }, 5}=\left( \textbf{A}_{\theta }\mid \textbf{E}_{\theta } \right) \textbf{k}_{\textbf{A}_{\theta },5}$$.
3. Next, it fetches $u_{A_{\theta }, \alpha , 2}$ and $u_{A_{\theta }, 4}$, and samples
  $$\begin{aligned} \textbf{k}_{\textbf{A}_{\theta }, \alpha , 2} \leftarrow \text{ SampleLeft }\left( \textbf{A}_{\theta }, \textbf{D}_{\theta }+\textbf{H}_{t}, \textbf{T}_{\textbf{A}_{\mathbf {\theta }}}, u'_{A_{\theta },\alpha , 2} \right) \end{aligned}$$
  where $$\alpha \in \text {KUNodes}(st,rl,t)$$, $$u'_{A_{\theta },\alpha , 2}=u_{A_{\theta },\alpha , 2}-u_{A_{\theta }, 4}r_{t}+u_{A_{\theta }, 5}$$ and $$\textbf{H}_{t} = Tran_{M\rightarrow V}\left( Tran_{V\rightarrow M} \left( \textbf{y}^{\top } \right) \textrm{H} \left( t \right) \right)$$.
4. Finally, it outputs the key update
  $$\begin{aligned} ku_{\theta ,t}=\left( \left\{ \textbf{k}_{\textbf{A}_{\theta }, \alpha , 2} \right\} _{\alpha \in \text {KUNodes}(st,rl,t)},\textbf{k}_{\textbf{A}_{\theta }, 5},r_{t} \right) . \end{aligned}$$

$\text {DKGen} \left( \{sk_{\theta }, ku_{\theta }\}_{\theta \in N} \right) \rightarrow dk$: The algorithm takes the secret key $\{sk_{\theta }\}_{\theta \in N}$ and the key update $\{ku_{\theta }\}_{\theta \in N}$ as input. If $Path(id)\cap \text {KUNodes}(st,rl,t)=\emptyset$, it returns a failure symbol $\perp$. Otherwise, it can find a unique node $\alpha \in Path(id) \cap \text {KUNodes}(st,rl,t)$ and store $\left( \textbf{k}_{\textbf{A}_{\theta },\alpha , 1}, \textbf{k}_{\textbf{A}_{\theta }, \alpha , 2}, \textbf{k}_{\theta , i} \right)$ in $dk_{\theta }$ for ench $\theta \in N$. For simplicity, we can omit the subscript $\alpha$. Then, let $\textbf{dk}_{\textbf{A}_{\theta }, 1}= \textbf{k}_{\textbf{A}_{\theta },1}+\textbf{k}_{\textbf{A}_{\theta },4}r_{t}$, $\textbf{dk}_{\textbf{A}_{\theta },2}= \textbf{k}_{\textbf{A}_{\theta },2}$, and $\textbf{dk}_{\textbf{A}_{\theta },3}= \textbf{k}_{\textbf{A}_{\theta },3}r_{t}-\textbf{k}_{\textbf{A}_{\theta },5}$, Finally, it outputs the decryption key

$$\begin{aligned} dk=\left\{ \textbf{dk}_{\textbf{A}_{\theta },1}, \textbf{dk}_{\textbf{A}_{\theta },2},\textbf{dk}_{\textbf{A}_{\theta },3}, \left\{ \textbf{k}_{\theta , i} \right\} _{i\in S} \right\} _{\theta \in N}. \end{aligned}$$

$\text {Enc} \left( pp, \{pk_{\theta }\}_{\theta \in N}, \mu ,\mathbb {W} \right) \rightarrow CT$: Given the public parameters *pp*, the public key $\{pk_{\theta }\}_{\theta \in N}$, a message $\mu$, and an access structure $\mathbb {W}=\left( \mathbb {W}^{+}\cap \mathbb {W}^{-} \right)$, which determines the set of positive and negative attributes, the algorithm samples $s\leftarrow \mathcal {R}_{q}$ and $e_{0}\leftarrow D_{\mathcal {R}_{q}, \sigma _{s}}$, and computes $\textbf{C}_{0} \leftarrow s \beta +e_{0}+\mu [q / 2]$. Then it samples vectors $\left\{ \textbf{e}_{\textbf{A}_{\theta }} \right\} _{\theta \in N}\leftarrow D_{\mathcal {R}_{q}^{m}, \sigma _{s}}$, and computes $\textbf{C}_{\textbf{A}_{\theta }} \leftarrow \textbf{A}_{\theta }^{T}s+\textbf{e}_{\textbf{A}_{\theta }}$. For each $\theta \in N$ and $i \in l_{\theta }$, if $i \in \mathbb {W}^{+}$, it samples vectors $\textbf{e}_{\theta ,i}\leftarrow D_{\mathcal {R}_{q}^{m}, \sigma _{s}}$ and computes $\textbf{C}_{\theta , i} \leftarrow \left( \textbf{B}_{\theta ,i}^{+}\right) ^{T}s+\textbf{e}_{\theta , i}$, else if $i \in \mathbb {W}^{-}$, it samples vectors $\textbf{e}_{\theta ,i}\leftarrow D_{\mathcal {R}_{q}^{m}, \sigma _{s}}$ and computes $\textbf{C}_{\theta , i} \leftarrow \left( \textbf{B}_{\theta ,i}^{-}\right) ^{T}s+\textbf{e}_{\theta , i}$. Otherwise, it samples vectors $\left\{ \textbf{e}_{\theta ,i}^{+},\textbf{e}_{\theta ,i}^{-} \right\} \leftarrow D_{\mathcal {R}_{q}^{m}, \sigma _{s}}$, and computes $\textbf{C}_{\theta , i}^{+} \leftarrow \left( \textbf{B}_{\theta ,i}^{+}\right) ^{T}s+\textbf{e}_{\theta , i}^{+}$ and $\textbf{C}_{\theta , i}^{-} \leftarrow \left( \textbf{B}_{\theta ,i}^{-}\right) ^{T}s+\textbf{e}_{\theta , i}^{-}$. Next, it samples matrices $\left\{ \mathbf {R_{\theta ,1}},\mathbf {R_{\theta ,2}} \right\} \leftarrow \left\{ \pm 1 \right\} ^{m\times m}$, and computes $\textbf{C}_{\theta , t,1}=\left( \textbf{D}_{\theta }+\textbf{H}_{t}\right) ^{T} s+\textbf{R}_{\theta ,1} \textbf{e}_{\textbf{A}_{\theta }}$ and $\textbf{C}_{\theta ,t,2}=\left( \textbf{E}_{\theta }\right) ^{T} s+\textbf{R}_{\theta ,2} \textbf{e}_{\textbf{A}_{\theta }}$. Finally, it outputs the ciphertext

$$\begin{aligned} CT =\left( \textbf{C}_{0}, \left\{ \textbf{C}_{\textbf{A}_{\theta }}, \textbf{C}_{\theta ,t,1}, \textbf{C}_{\theta ,t,2}\right\} _{\theta \in N},\left\{ \textbf{C}_{\theta , i}, \textbf{C}_{\theta , i}^{\pm }\right\} _{\theta \in N,i\in l_{\theta }}\right) . \end{aligned}$$

$\text {Dec} \left( CT, dk \right) \rightarrow \mu$: The algorithm takes the ciphertext *CT* and the decryption key *dk* as input. Let $\textbf{S}$ be the attribute set associated to *dk*. If $\textbf{S}\cap \mathbb {W}^{+}=\mathbb {W}^{+}$ and $\textbf{S}\cap \mathbb {W}^{-}=\emptyset$, it proceeds as follows:

1. Calculate $$a_{\theta ,1}=\left( \textbf{C}_{\textbf{A}_{\theta }} \right) ^{\top }\textbf{dk}_{\textbf{A}_{\theta },1}$$.
2. For each $\theta \in N$ and $i \in l_{\theta }$, if $i \in \mathbb {W}$, calculate $a_{\theta , 2, i}=\left( \textbf{C}_{\theta , i} \right) ^{\top }\textbf{k}_{\theta , i}$, else if $i \in S$, calculate $a_{\theta , 2, i}=\left( \textbf{C}_{\theta , i}^{+} \right) ^{\top }\textbf{k}_{\theta , i}$, otherwise, calculate $a_{\theta , 2, i}=\left( \textbf{C}_{\theta , i}^{-} \right) ^{\top }\textbf{k}_{\theta , i}$.
3. Let $\textbf{C}_{\textbf{A}_{\theta },t,1}=\left( \textbf{A}_{\theta }\mid \textbf{D}_{\theta }+\textbf{H}_{t}\right) ^{T} s+\left( \textbf{e}_{\textbf{A}_{\theta }}\mid \textbf{R}_{\theta ,1 } \textbf{e}_{\textbf{A}_{\theta }} \right)$, and calculate $a_{\theta ,3}=\textbf{C}_{\textbf{A}_{\theta }, t}\textbf{dk}_{\textbf{A}_{\theta },2}$.
4. Let $\textbf{C}_{\textbf{A}_{\theta },t,2}=\left( \textbf{A}_{\theta }\mid \textbf{E}_{\theta }\right) ^{T} s+\left( \textbf{e}_{\textbf{A}_{\theta }}\mid \textbf{R}_{\theta ,2 } \textbf{e}_{\textbf{A}_{\theta }} \right)$, and calculate $a_{\theta ,4}=\textbf{C}_{\textbf{A}_{\theta }, t}\textbf{dk}_{\textbf{A}_{\theta },3}$.
5. Calculate
  $$\begin{aligned} t=\textbf{C}_{0}-\sum _{\theta \in N}\mathcal {L}_{\theta }\left( a_{\theta ,1}+a_{\theta ,2}+a_{\theta ,3}+a_{\theta ,4} \right) \end{aligned}$$
  where $a_{\theta ,2}=\sum _{i \in l_{\theta }}a_{\theta ,2,i}$, and $\mathcal {L}_{\theta }=\frac{\Pi _{\theta \in N,\theta \ne \delta }\left( -\theta \right) }{\Pi _{\theta \in N,\theta \ne \delta }\left( \delta -\theta \right) }$ is the Lagrangian coefficient.
6. If $\left| t_{i} \right| < \frac{q}{4}$, output $\mu _{i}=0$, otherwise output $\mu _{i}=1$.

Otherwise, it outputs a failure symbol $\perp$.

$\text {Rev} \left( rl, id, t \right) \rightarrow rl$: The algorithm takes as input the revocation list *rl*, an user’s global identity *id* to be revoked and a time *t*. It updates revocation list *rl*

$$\begin{aligned} rl \leftarrow rl \cup \left( id,t \right). \end{aligned}$$

### Correctness

We assume that an attribute set $\textbf{S}$ satisfies the access policy($\textbf{S}\cap \mathbb {W}^{+}=\mathbb {W}^{+}$ and $\textbf{S}\cap \mathbb {W}^{-}=\emptyset$), then we have

$$\begin{aligned}{} & {} a_{\theta ,1}+ a_{\theta ,2}+a_{\theta ,3}+a_{\theta ,4}\\= & {} \left( \textbf{C}_{\textbf{A}_{\theta }} \right) ^{\top } \textbf{dk}_{\textbf{A}_{\theta },1}+\sum _{i \in l_{\theta }}\left( \textbf{C}_{\theta , i}^{*} \right) ^{\top } \textbf{k}_{\theta , i} +\left( \textbf{C}_{\textbf{A}_{\theta },t,1} \right) ^{\top } \textbf{dk}_{\textbf{A}_{\theta },2} \\{} & {} +\left( \textbf{C}_{\textbf{A}_{\theta },t,2} \right) ^{\top } \textbf{dk}_{\textbf{A}_{\theta },3} \\= & {} s\textbf{A}_{\theta }\textbf{dk}_{\textbf{A}_{\theta },1} + \sum _{i \in l_{\theta }}s\textbf{B}_{\theta ,i}^{*}\textbf{k}_{\theta ,i} + s\left( \textbf{A}_{\theta }\mid \textbf{D}_{\theta } +\textbf{H}_{t}\right) \textbf{dk}_{\textbf{A}_{\theta },2} \\{} & {} +s\left( \textbf{A}_{\theta }\mid \textbf{E}_{\theta } \right) \textbf{dk}_{\textbf{A}_{\theta },3} +\textbf{e}_{\textbf{A}_{\theta }}^{\top }\textbf{dk}_{\textbf{A}_{\theta },1} + \textbf{e}_{\theta ,i}^{\top }\textbf{k}_{\theta ,i}\\{} & {} +\left( \textbf{e}_{\textbf{A}_{\theta }}\mid \textbf{R}_{\theta ,1} \textbf{e}_{\textbf{A}_{\theta }}\right) ^{\top }\textbf{dk}_{\textbf{A}_{\theta },2}+\left( \textbf{e}_{\textbf{A}_{\theta }}\mid \textbf{R}_{\theta ,2} \textbf{e}_{\textbf{A}_{\theta }}\right) ^{\top }\textbf{dk}_{\textbf{A}_{\theta },3}\\= & {} s\textbf{A}_{\theta }\textbf{k}_{\textbf{A}_{\theta },1} + s\textbf{A}_{\theta }\textbf{k}_{\textbf{A}_{\theta },4}r_{t} + \sum _{i \in l_{\theta }}s\textbf{B}_{\theta ,i}^{*}\textbf{k}_{\theta ,i} + s\left( \textbf{A}_{\theta }\mid \textbf{E}_{\theta } \right) \textbf{k}_{\textbf{A}_{\theta },3}r_{t} \\{} & {} +s\left( \textbf{A}_{\theta }\mid \textbf{D}_{\theta } +\textbf{H}_{t}\right) \textbf{k}_{\textbf{A}_{\theta },2} - s\left( \textbf{A}_{\theta }\mid \textbf{E}_{\theta } \right) \textbf{k}_{\textbf{A}_{\theta },5}+\mathbf {\widetilde{e}} \\= & {} su_{A_{\theta },1} +\left( su_{A_{\theta },4}-su_{A_{\theta },3} \right) r_{t} +\sum _{i \in l_{\theta }}su_{\theta ,i} +s\left( u'_{A_{\theta },\alpha , 2} \right) \\{} & {} +su_{A_{\theta },3}r_{t} - su_{A_{\theta }, 5}+\mathbf {\widetilde{e}}_{\theta } \\= & {} s\beta _{\theta } + \mathbf {\widetilde{e}}_{\theta } \end{aligned}$$

where, if $i\in \mathbb {W}$, $\textbf{C}_{\theta ,i}^{*} = \textbf{C}_{\theta ,i}$, else if $i\in S$, $\textbf{C}_{\theta ,i}^{*} = \textbf{C}^{+}_{\theta ,i}$, otherwise, $\textbf{C}_{\theta ,i}^{*} = \textbf{C}^{-}_{\theta ,i}$, and the choice of $\textbf{B}^{*}_{\theta ,i}$ corresponds to that of $\textbf{C}_{\theta ,i}^{*}$. Furthermore, the total noise term is denoted by $\mathbf {\widetilde{e}}_{\theta } = \textbf{e}_{\textbf{A}_{\theta }}^{\top }\textbf{k}_{\textbf{A}_{\theta },1}+\textbf{e}_{\textbf{A}_{\theta }}^{\top }\textbf{k}_{\textbf{A}_{\theta },4}r_{t} + \textbf{e}_{\theta ,i}^{\top }\textbf{k}_{\theta ,i} + \left( \textbf{e}_{\textbf{A}_{\theta }}\mid \textbf{R}_{\theta ,1} \textbf{e}_{\textbf{A}_{\theta }}\right) ^{\top }\textbf{k}_{\textbf{A}_{\theta },2}+\left( \textbf{e}_{\textbf{A}_{\theta }}\mid \textbf{R}_{\theta ,2} \textbf{e}_{\textbf{A}_{\theta }}\right) ^{\top }\left( \textbf{k}_{\textbf{A}_{\theta },3}r_{t}+\textbf{k}_{\textbf{A}_{\theta },5} \right)$. Next, for $\forall \theta \in N$, we compute

$$\begin{aligned}{} & {} \textbf{C}_{0}-\sum _{\theta \in N}\mathcal {L}_{\theta }\left( a_{\theta ,1}+ a_{\theta ,2}+a_{\theta ,3}+a_{\theta ,4} \right) \\= & {} s\beta +e_{0}+\mu [q / 2]-\sum _{\theta \in N}\mathcal {L}_{\theta }\left( s\beta _{\theta } + \mathbf {\widetilde{e}}_{\theta }\right) \\= & {} \mu [q / 2]+e_{0} - \sum _{\theta \in N}\mathcal {L}_{\theta }\mathbf {\widetilde{e}}_{\theta }. \end{aligned}$$

When the noise is small enough, it will not affect the plaintext information in the ciphertext. We let the upper bound on the combination of all noise factors be $\eta$, the upper bounds of key components $\left\{ \textbf{k}_{\textbf{A}_{\theta },1}, \textbf{k}_{\textbf{A}_{\theta },2},\textbf{k}_{\textbf{A}_{\theta },3},\textbf{k}_{\textbf{A}_{\theta },4},\textbf{k}_{\textbf{A}_{\theta },5}, \textbf{k}_{\theta ,i} \right\}$ are $\eta _{s}$, and the upper bounds of ciphertext noise factors $\textbf{e}_{\textbf{A}_{\theta }}$, $\textbf{e}_{\theta ,i}$ are $\eta _{e}$. According to [19], we let $\eta _{e} =8\sigma$, and $\eta _{s} =8\sigma _{s}$. In order to ensure the correctness of decryption, the following inequality holds with non-negligible probability, i.e.,

$$\begin{aligned} \eta = \eta _{e}\eta _{s}\sqrt{nm\left( l+(3+2\eta _{e})N \right) } < \frac{q}{4}, \end{aligned}$$

where *l* is the sum of the number of attributes in all attribute authorities. Finally, we have that

$$\begin{aligned} q>256\sigma \sigma _{s}\sqrt{nm\left( l+(3+16\sigma )N \right) }. \end{aligned}$$

### Security proof

#### Theorem 1

If the RLWE assumption holds, then our scheme is secure against the selective IND-CPA described in Security model section.

In the security proof, we divided the adversary into two types, one is the adversary who has not obtained legal authorization, and the user’s private key they have does not satisfies the challenge access policy $\mathbb {W}^{*}$. The other category is revoked users who indicate malicious intent. Adversary can query the user’s private key that satisfies the challenge access policy and the updated key and decryption key before the revoked time *t* $(t<t^{*})$. To prove the above theorem, we will describe several security games that differ from each other in the formation of public parameters, the key queried by adversary $\mathcal {A}$ and the challenge ciphertext. The first game is the same as the ABE game we defined in the security model, and the adversary’s advantage is zero in the last game of the sequence. And we argue that the adversary $\mathcal {A}$’s advantage varies negligibly between each successive security game. This will prove that the adversary has a negligible advantage in winning the original ABE security game.

$\textbf{Game}_{\varvec{0}}$: This is the real selective security game form Security model section between an adversary $\mathcal {A}$ against our scheme and a RM-CP-ABE challenger $\mathcal {B}$.

$\textbf{Game}_{\varvec{1}}$: This game is the same as the previous game except the way the public key vectors $\left\{ \textbf{A}_{\theta }, \textbf{B}_{\theta ,i}^{\pm },\textbf{D}_{\theta },\right\}$ are generated for all $\theta \in N$ and $i\in l_{\theta }$ during the setup phase. In this game, the public key vectors $\left\{ \textbf{A}_{\theta } \right\} _{\theta \in N}$ is uniformly randomly chosen over $\mathcal {R}_{q}^{1 \times m}$ instead of by the $\text {TrapGen}$ algorithm. For all $\theta \in N$ and $i \in l_{\theta }\setminus \mathbb {W}^{*}$, $\mathcal {B}$ samples $\left\{ \textbf{B}^{+}_{\theta ,i},\textbf{B}^{-}_{\theta ,i} \right\} \leftarrow \mathcal {R}_{q}^{1\times m}$. For each $i \in \mathbb {W}^{+}$, $\mathcal {B}$ samples $\textbf{B}^{+}_{\theta ,i}\leftarrow \mathcal {R}_{q}^{1\times m}$ and computes $\left\{ \textbf{B}^{-}_{\theta ,i},\textbf{T}_{\textbf{B}_{\theta ,i}}^{-} \right\} \leftarrow \text {TrapGen}(\lambda )$. Correspondingly, $\mathcal {B}$ samples $\textbf{B}^{-}_{\theta ,i}\leftarrow \mathcal {R}_{q}^{1\times m}$ and computes $\left\{ \textbf{B}^{+}_{\theta ,i},\textbf{T}_{\textbf{B}_{\theta ,i}}^{+} \right\} \leftarrow \text {TrapGen}(\lambda )$ for each $i \in \mathbb {W}^{-}$. Then, $\mathcal {B}$ samples $\left\{ \textbf{R}_{\theta ,1},\textbf{R}_{\theta ,2} \right\} \leftarrow \left\{ -1,1 \right\} ^{m\times m}$ for each $\theta \in N$. Next, it computes $\textbf{D}_{\theta }\leftarrow \textbf{A}_{\theta }\textbf{R}_{\theta ,1}-\textbf{H}_{t^{*}}$ and $\textbf{E}_{\theta }\leftarrow \textbf{A}_{\theta }\textbf{R}_{\theta ,2}$. According to the properties of the $\text {TrapGen}$ algorithm and the leftover hash lemma, we conclude that $\textbf{Game}_{\varvec{0}}$ and $\textbf{Game}_{\varvec{1}}$ are statistically indistinguishable from the adversary’s view.

$\textbf{Game}_{\varvec{2}}$: This game is analogous to the previous game except the generationed of the public parameter $\textbf{y}$ during the setup phase. In this game, the vector $\textbf{y}$ is generated by $\text {TrapGen}(\lambda )$ instead of being randomly sampled. The indistinguishably between $\textbf{Game}_{\varvec{1}}$ and $\textbf{Game}_{\varvec{2}}$ follows from the good properties of the $\text {TrapGen}\left( \cdot \right)$ algorithm.

$\textbf{Game}_{\varvec{3}}$: In this game, we change the way the secret keys $\left\{ sk_{\theta } \right\} _{\theta \in N}$ and update keys $\left\{ ku_{\theta ,t} \right\} _{\theta \in N}$ are generated during the global setup phase. According to the key query restrictions on the two cases in the security model, we make the following changes:

${Case\ I}$: If the adversary $\mathcal {A}$ query an identity *id* whose attribute set $\textbf{S}$ does not satisfy the access policy $\mathbb {W^{*}}$, for all $\theta \in N$ and $t = t^{*}$, $\mathcal {B}$ chooses $\left\{ \textbf{k}_{\textbf{A}_{\theta }, \alpha , 2},\textbf{k}_{\textbf{A}_{\theta },4} \right\} \leftarrow \mathcal {R}_{q}^{ m}$ and computes $u_{A_{\theta }, 4}=\textbf{A}_{\theta }\textbf{k}_{\textbf{A}_{\theta }, 4}+u_{A_{\theta }, 3}$. Then it computes $u_{A_{\theta }, \alpha , 2}= \left( \textbf{A}_{\theta }\mid \textbf{A}_{\theta }\textbf{R}_{\theta ,1}\right) \textbf{k}_{\textbf{A}_{\theta }, \alpha , 2}+u_{A_{\theta }, 4}r_{t^{*}}-u_{A_{\theta }, 5}$ and store them in node. Next, for any $t \ne t^{*}$, $\mathcal {B}$ samples $\textbf{k}_{\textbf{A}_{\theta }, \alpha , 2} \leftarrow \text{ SampleRight } (\textbf{A}_{\theta }, \textbf{A}_{\theta }\textbf{R}_{\theta ,1} - \textbf{H}_{t^{*}} + \textbf{H}_{t}, \textbf{T}_{\textbf{A}_{\varvec{\theta }}}, u_{A_{\theta },\alpha , 2}-u_{A_{\theta }, 4}\left( r_{t}-r_{t^{*}}\right) +u_{A_{\theta }, 5})$. For secret key query, $\mathcal {B}$ chooses $\textbf{k}_{\textbf{A}_{\theta }, \alpha , 1}\leftarrow \mathcal {R}_{q}^{1\times m}$ and computes $u_{A_{\theta }, \alpha , 1}=\textbf{A}_{\theta }\textbf{k}_{\textbf{A}_{\theta }, \alpha , 1}$ for all $\theta \in N$. Since $\textbf{S}$ does not satisfy $\mathbb {W^{*}}$, $\mathcal {B}$ must knows at least one $\textbf{T}_{\textbf{B}_{\theta ,j}}^{+}$ or $\textbf{T}_{\textbf{B}_{\theta ,j}}^{-}$. Then, $\mathcal {B}$ calculates $u_{\theta , i} \leftarrow \textbf{B}_{\theta ,i}^{-} \textbf{k}_{\theta , i}$ for each $i \in \textbf{S},\,i \ne j$ and samples $\textbf{k}_{\theta , j} \leftarrow \text{ SamplePre }\left( \textbf{B}_{\theta ,j}^{*}, \textbf{T}_{\textbf{B}_{\theta ,\textbf{j}}}^{*}, u_{\theta , j}, \sigma , \sigma _{s}\right)$, where $u_{\theta , j}=\beta _{\theta }-u_{A_{\theta }, \alpha , 1}-u_{A_{\theta , \alpha , 2}}-\sum _{i \in l_{\theta },i \ne j} u_{\theta , i}$.

${Case\ II}$: If the adversary $\mathcal {A}$ query an identity *id* whose attribute set $\textbf{S}$ does satisfy the access policy $\mathbb {W^{*}}$, then the identity *id* have been revoked before a time $t^{*}$. For each $\theta \in N$ and $\alpha \in Path(id) \cap \text {KUNodes}(st,rl,t)$, $\mathcal {B}$ chooses $\textbf{k}_{\textbf{A}_{\theta }, \alpha , 1}\leftarrow \mathcal {R}_{q}^{m}$ and computes $u_{A_{\theta }, \alpha , 1}=\textbf{A}_{\theta }\textbf{k}_{\textbf{A}_{\theta }, \alpha , 1}$. Then, $\mathcal {B}$ computes $u_{A_{\theta , \alpha , 2}}=\beta _{\theta }-u_{A_{\theta }, \alpha , 1}-\sum _{i \in l_{\theta }} u_{\theta , i}$ and store them in the node.

The indistinguishably between $\textbf{Game}_{\varvec{2}}$ and $\textbf{Game}_{\varvec{3}}$ follows from the good sampling property of $\text {SamplePre}\left( \cdot \right)$ algorithm.

$\textbf{Game}_{\varvec{4}}$: This game is identical to $\textbf{Game}_{\varvec{3}}$, except that the challenge ciphertext is generated. In this game, for all $\theta \in N,\,i \in l_{\theta }\setminus \mathbb {W}^{*}$, the challenger $\mathcal {B}$ sets $\left\{ \textbf{C}_{\textbf{A}_{\theta }},\textbf{C}_{\theta ,i},\textbf{C}_{\theta ,i}^{\pm } \right\} \leftarrow \mathcal {R}_{q}^{m}$, $\textbf{C}_{\theta ,t,1}=\textbf{R}_{\theta ,1}\textbf{C}_{\textbf{A}_{\theta }}$, and $\textbf{C}_{\theta ,t,2}=\textbf{R}_{\theta ,2}\textbf{C}_{\textbf{A}_{\theta }}$. The indistinguishably between $\textbf{Game}_{\varvec{3}}$ and $\textbf{Game}_{\varvec{4}}$ follows from the RLWE problem. Since the challenge ciphertext is a random element in the ciphertext space, the advantage of adversary $\mathcal {A}$ in this game is negligible.

## Efficiency analysis

In this section, we carry out theoretical analysis and simulation implementation of our scheme. In terms of theoretical analysis, we compare our scheme and related work in terms of scheme characteristics, storage cost, and computational cost. In the experimental simulation, we focus on analyzing the time cost of each algorithm when the number of attributes is different.

### Theoretical analysis

In this subsection, we gave a theoretical analysis in functions of schemes, storage cost, and computational cost by comparing related schemes in [8, 9, 12, 13, 17, 18, 25] with our scheme.

Table 1 shows the comparison of related schemes and our scheme in terms of security assumptions and the scheme’s functions. Observe that schemes in [9, 17, 18, 25] only support single attribute authority. While schemes in [8, 12, 13] and our scheme support multi-attribute authority, which can effectively reduce the computational burden of each attribute authority. In addition, the schemes in [8, 9, 25] and our scheme also support user revocation, allowing the system to manage users dynamically, improving the practicability of the scheme. On this basis, the scheme in [25] and our scheme also considers more possible security issues with decryption key exposure resistance.

**Table 1:** Scheme Functions

Scheme	Model	Multi-authority	Revocable	DKER	Assumption
Scheme [17]	CP-ABE	$\times$	$\times$	$\times$	LWE
Scheme [13]	CP-ABE	$\checkmark$	$\times$	$\times$	LWE
Scheme [12]	CP-ABE	$\checkmark$	$\times$	$\times$	LWE
Scheme [9]	CP-ABE	$\times$	$\checkmark$	$\times$	LWE
Scheme [25]	KP-ABE	$\times$	$\checkmark$	$\checkmark$	LWE
Scheme [18]	CP-ABE	$\times$	$\times$	$\times$	RLWE
Scheme [8]	CP-ABE	$\checkmark$	$\checkmark$	$\times$	RLWE
Our Scheme	CP-ABE	$\checkmark$	$\checkmark$	$\checkmark$	RLWE

Table 2 shows the storage cost of related schemes and our scheme in terms of public parameter size, private key size, and ciphertext size. For the convenience of description, we let *N* denote the number of attribute authorities in the system, $\left| l_{s} \right|$ denote the total number of attributes in the system, $\left| l_{a} \right|$ denote the number of attributes in the access policy, and $\left| l_{u} \right|$ denote the number of attributes owned by users. It is worth mentioning that schemes in [9, 12, 13, 17, 25] are constructed based on the LWE assumption, where the public parameter $m=\Omega \left( n\log q \right)$, while the scheme in [8, 18] and our scheme are constructed based on the RLWE assumption. It is well-known that the scheme based on RLWE is usually superior to the schemes based on LWE in the parameter size and efficiency. Therefore, the scheme in [8, 18] and our scheme have smaller parameters than schemes [9, 12, 13, 17, 25], and are more efficient.

**Table 2:** Storage cost comparison

Scheme	Public parameter size	Decryption key size	Ciphertext size
Scheme [17]	$(2\left| l_{s} \right| +2)mn\left\lceil \log q \right\rceil$	$2(\left| l_{u} \right| +\left| l_{s} \right| )mn\left\lceil \log q \right\rceil$	$\left( 2\left| l_{a} \right| +2\left| l_{s} \right| +1 \right) mn\left\lceil \log q \right\rceil$
Scheme [13]	$\left| l_{s} \right| (m+2)n \left\lceil \log q \right\rceil$	$\left| l_{u} \right| m\left\lceil \log q \right\rceil$	$\left( \left| l_{a} \right| +1 \right) m\left\lceil \log q \right\rceil$
Scheme [12]	$2\left| l_{s} \right| mn\left\lceil \log q \right\rceil$	$2\left| l_{u} \right| m\left\lceil \log q \right\rceil$	$\left( 2\left| l_{a} \right| +1 \right) m\left\lceil \log q \right\rceil$
Scheme [9]	$5\left| l_{s} \right| \left( m+1 \right) n\left\lceil \log q \right\rceil$	$4\left| l_{s} \right| m \left\lceil \log q \right\rceil$	$( 3\left| l_{s} \right| m +1 ) \left\lceil \log q \right\rceil$
Scheme [25]	$\left( \left| l_{s} \right| +3 \right) mn\left\lceil \log q \right\rceil$	$3mn\left\lceil \log q \right\rceil$	$\left( \left| l_{s} \right| +2 \right) m\left\lceil \log q \right\rceil$
Scheme [18]	$(2\left| l_{s} \right| +1)mn\left\lceil \log q \right\rceil$	$(\left| l_{u} \right| +\left| l_{s} \right| )mn\left\lceil \log q \right\rceil$	$\left( 2\left| l_{a} \right| +2\left| l_{s} \right| \right) mn\left\lceil \log q \right\rceil$
Scheme [8]	$(4\left| l_{s} \right| +N+2)mn\left\lceil \log q \right\rceil$	$4(\left| l_{u} \right| +\left| l_{s} \right| )mn\left\lceil \log q \right\rceil$	$\left( 2\left| l_{a} \right| +2\left| l_{s} \right| +1 \right) mn\left\lceil \log q \right\rceil$
Our Scheme	$\left( \left| l_{s} \right| +3N+1 \right) mn\left\lceil \log q \right\rceil$	$\left( \left| l_{s} \right| +3N \right) mn\left\lceil \log q \right\rceil$	$\le \left( 2\left| l_{s} \right| +2N \right) mn\left\lceil \log q \right\rceil$

### Experimental simulation

#### Test environment

We have implemented our construction on an Ubuntu 18.04 operating system with Intel Core i5-10400F, 2.90GHz processor and 4GB of memory with the Palisade library.

#### Storage cost

In our implementation, we use the Gaussian sampling algorithm for rings in scheme [19]. Specifically, we set the base $b=64$, the ring size $n=1024$ and the number of attribute authority $N=3$. Table 3 shows the storage overhead of our scheme under the above parameter settings and different number of attributes. Note that the ciphertext size of our scheme will change according to $\left| l_{a} \right|$. When $\left| l_{a} \right| =1$, the ciphertext size reaches the maximum value, the formula for ciphertext size (in number of bits) can be given as $\left( 2\left| l_{s} \right| +2N \right) \cdot m\cdot n\cdot \left\lceil \log q \right\rceil$. When $\left| l_{a} \right| =\left| l_{s} \right|$, the ciphertext size reaches the minimum value, the formula can be given as $\left( \left| l_{s} \right| +2N+1 \right) \cdot m\cdot n\cdot \left\lceil \log q \right\rceil$. Compared with the schemes based on the LWE assumption, our scheme has a relatively small cost in terms of storage. Moreover, as the number of attributes in the system increases, the public key size, decryption key size and ciphertext size of our scheme increase slowly. Therefore, our scheme is completely feasible in practical scenarios.

**Table 3:** Storage cost of our scheme

$(l_{s},l_{a})$	Public parameter size	Decryption key size	Ciphertext size
(6, 1)	2.67 MB	2.5 MB	3 MB
(6, 6)	2.67 MB	2.5 MB	2.17 MB
(12, 1)	3.67 MB	3.5 MB	6 MB
(12, 12)	3.67 MB	3.5 MB	3.17 MB
(18, 1)	4.67 MB	4.5 MB	8 MB
(18, 18)	4.67 MB	4.5 MB	4.17 MB

#### Situation results

**Fig. 4:**
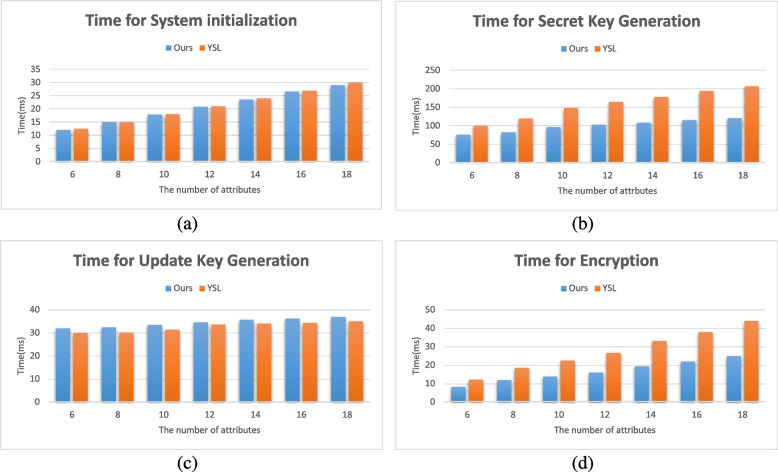
Time cost of scheme

Figure 4 shows the time cost comparison between recent work [8] (refer to YSL) and our scheme in initialization phase, private key generation phase, update key generation phase and encryption phase. We set the number of attributes vary from 6 to 18 in an increment of 2, hence there are 7 different situations. For each case, the experiment was repeated 20 times and each experiment was completely independent, and finally the average value was taken as the experimental result. In Fig. 4a, since YSL and our scheme both support multiple attribute authorities to work together, the time cost in the system initialization phase is both small. And both increase linearly with the increase of the number of attributes, and the growth rate is about 1.42 (ms/item). Therefore, even in a system with a large number of attributes, the setup phase time cost is acceptable. Figure 4b shows the time cost of YSL and our scheme in terms of user private key generation. Since our scheme has smaller user key size and number of key components than YSL, it takes less time. And as the number of attributes increases, the time cost of our scheme grows more slowly. In Fig. 4c, YSL has a slightly smaller time cost in updating key generation than our scheme. However, the efficiency of update key generation is related to the number of users in the system and not to the number of attributes. In YSL, the time cost of generating updated keys is linear with the number of users in the system, while the time cost of our scheme grows logarithmically with the number of users in the system. Therefore, in the case of a large number of attributes and users in the system, our update key generation algorithm can still maintain a high efficiency. Figure 4d shows the time cost comparison between YSL and our scheme in terms of encryption. During the encryption process, our scheme operates more compactly, and the number of ciphertext components associated with each attribute is also less than YSL. Therefore, the time cost of our scheme in the encryption phase is less than that of YSL, and as the number of attributes increases, the time cost of our scheme increases more slowly.

**Fig. 5:**
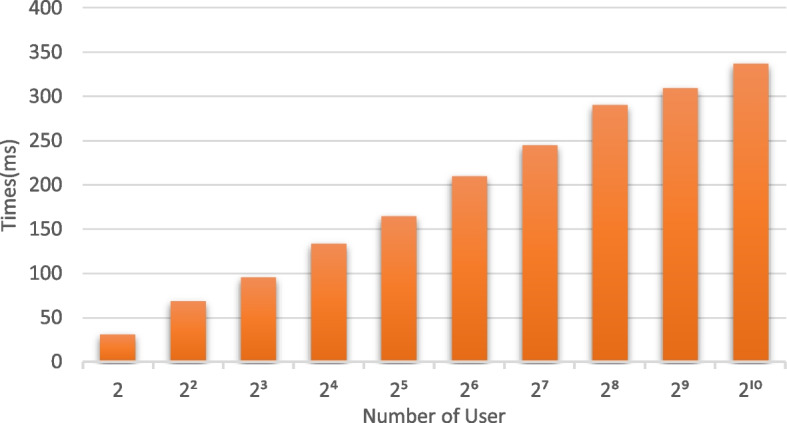
Time cost of KeyUp algorithm

Figure 5 shows the relationship between the number of users in the system and the generation time of the update key. We denote the number of users by $N_{u}$. When no user in the system has been revoked, all users share a set of update keys. At this time, the attribute authority only needs to generate a set of update keys. When a user is revoked, the attribute authority needs to generate $\left\lceil \log N_{u} \right\rceil$ update key components. Observe that the time cost of updating the key generation algorithm grows linearly and slowly when the number of users in the system grows exponentially. Therefore, our scheme is also suitable for large systems with a large number of users.

## Conclusion

We propose an RM-CP-ABE scheme suitable for CFS systems. It implements efficient dynamic management of users and supports distributed frameworks. In addition, it is also resistant to decryption key exposure attack. By using the game sequence, we prove the security against CPA attacks and collusion attacks under the random oracle model. We also conducted an implementation to demonstrate the practicability of our RLWE-based RM-CP-ABE scheme. The future works will focus on the construction of secure and efficient CP-ABE scheme under the standard model. At the same time, we can also consider constructing a CP-ABE scheme with more flexible access policies.

## Data Availability

The datasets generated during and/or analyzed during the current study are available from the corresponding author on reasonable request.
